# [Retracted] Tanshinone II A stabilizes vulnerable plaques by suppressing RAGE signaling and NF‑κB activation in apolipoprotein‑E‑deficient mice

**DOI:** 10.3892/mmr.2024.13410

**Published:** 2024-12-02

**Authors:** Dong Zhao, Lufang Tong, Lixin Zhang, Hong Li, Yingxin Wan, Tiezhong Zhang

Mol Med Rep 14: 4983–4990, 2016; DOI: 10.3892/mmr.2016.5916

Following the publication of this paper, it was drawn to the Editor's attention by a concerned reader that certain of the western blotting data shown in Fig. 4B on p. 4988 had already appeared in an article written by different authors at different research institutes that had already been published. In addition, it appeared as if some of the control β-actin protein bands had been re-used in Fig. 3 on p. 4987, comparing between Fig. 3A and C.

Owing to the fact that the contentious data in the above article had already been published prior to its submission to *Molecular Medicine Reports*, the Editor has decided that this paper should be retracted from the Journal. The authors were asked for an explanation to account for these concerns, but the Editorial Office did not receive a reply. The Editor apologizes to the readership for any inconvenience caused.

